# Chalcones Enhance TRAIL-Induced Apoptosis in Prostate Cancer Cells

**DOI:** 10.3390/ijms11010001

**Published:** 2009-12-24

**Authors:** Ewelina Szliszka, Zenon P Czuba, Bogdan Mazur, Lukasz Sedek, Andrzej Paradysz, Wojciech Krol

**Affiliations:** 1 Chair and Department of Microbiology and Immunology, Medical University of Silesia in Katowice, Jordana 19, 41 808 Zabrze, Poland; E-Mails: eszliszka@sum.edu.pl (E.S.); zczuba@sum.edu.pl (Z.P.C.); bmazur@sum.edu.pl (B.M.); 2 Chair and Department of Pediatric Hematology and Oncology, Medical University of Silesia in Katowice, 3-go Maja 13, 41 800 Zabrze, Poland; E-Mail: lsedek@sum.edu.pl (L.S.); 3 Chair and Department of Urology, Medical University of Silesia in Katowice, 3-go Maja 13, 41 800 Zabrze, Poland; E-Mail: parady@poczta.onet.pl (A.P.)

**Keywords:** chalcones, TRAIL (tumor necrosis factor-related apoptosis-inducing ligand), apoptosis, chemoprevention, prostate cancer

## Abstract

Chalcones exhibit chemopreventive and antitumor effects. Tumor necrosis factor-related apoptosis-inducing ligand (TRAIL) is a naturally occurring anticancer agent that induces apoptosis in cancer cells and is not toxic to normal cells. We examined the cytotoxic and apoptotic effect of five chalcones in combination with TRAIL on prostate cancer cells. The cytotoxicity was evaluated by the MTT and LDH assays. The apoptosis was determined using flow cytometry with annexin V-FITC. Our study showed that all five tested chalcones: chalcone, licochalcone-A, isobavachalcone, xanthohumol, butein markedly augmented TRAIL-mediated apoptosis and cytotoxicity in prostate cancer cells and confirmed the significant role of chalcones in chemoprevention of prostate cancer.

## 1. Introduction

Chalcones (1,3-diphenyl-2-propen-1-ones)—one of the major classes of natural products with widespread distribution in spices, tea, beer, fruits and vegetables—have been recently subject of great interest for their pharmacological activities [1]. Chalcones are precursor compounds in flavonoid biosynthesis in plants. Chemically they consist of open-chain flavonoids in which the two aromatic rings are joined by a three-carbon *α,β*-unsaturated carbonyl system [2–4]. Chalcones have been reported to possess antiinflammatory, antimicrobial, antioxidant and anticancer properties [4–9].

Prostate cancer is one of the most commonly diagnosed cancers in men, and the second leading cause of cancer deaths in the European Union and United States of America [10]. Many antitumor drugs have been developed for prostate cancer patients, but their intolerable systemic toxicity often limits their clinical use. Chemoprevention is one of the most promising approaches in prostate cancer research, in which natural or synthetic agents are used to prevent this malignant disease [10–12]. The *in vitro* studies showed that chalcones inhibit the proliferation of prostate cancer cells by inducing apoptosis and blocking cell cycle progression [13–17].

Tumor necrosis factor related apoptosis inducing ligand (TRAIL), a member of TNF superfamily, selectively induces apoptosis in cancer cells with no toxicity against normal tissues. Soluble or expressed on lymphocytes T, macrophages and NK cells, molecules TRAIL plays an important role in immune surveillance and defense mechanisms against tumor cells. TRAIL induces programmed death in various cancer cells through its interaction with the death receptor TRAIL-R1 and/or TRAIL-R2 [18,19]. However, some tumor cells are resistant to TRAIL-mediated cytotoxicity. The decreased expression of death receptors TRAIL-R1 (DR4) and TRAIL-R2 (DR5) or increased expression of antiapoptotic proteins in cancer cells are involved in TRAIL-resistance [20]. We and others have shown that TRAIL-resistant prostate cancer cells can be sensitized by chemotherapeutic agents, ionizing radiation or dietary polyphenols [21–25].

In this work we investigated the apoptotic and/or cytotoxic effect of chalcones in combination with TRAIL on prostate cancer cells. Our results indicated that all five tested chalcones: chalcone, licochalcone-A, isobavachalcone, xanthohumol and butein markedly augment TRAIL mediated apoptosis in LNCaP prostate cancer cells. We showed for the first time that chalcones sensitize prostate cancer cells to TRAIL induced apoptosis. The TRAIL-mediated cytotoxic and apoptotic pathways may be a target of chemopreventive agents in prostate cancer cells and overcoming TRAIL-resistance by chalcones may be one of the mechanisms responsible for their cancer preventive effects.

## 2. Results and Discussion

### 2.1. Cytotoxic and Apoptotic Effects of Chalcones in Prostate Cancer Cells

**Figure 1.**
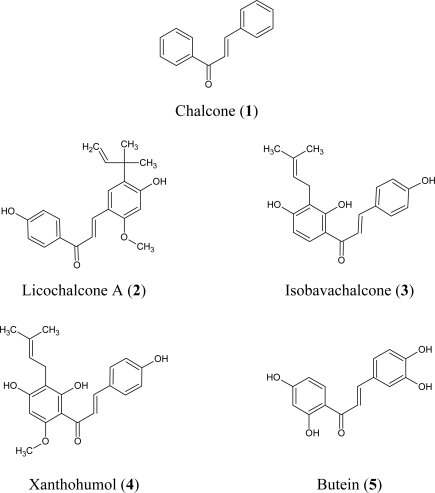
Chemical structures of the studied chalcones.

Recent epidemiological studies have confirmed the role of polyphenols in prevention of prostate cancer [11,26,27]. Chalcones are plant-derived polyphenols belonging to the flavonoid family and widely investigated in various therapeutic areas. The previous experimental studies suggested that these compounds exert *in vitro* chemopreventive activity [6–9]. Chalcones inhibit cell growth and induce apoptosis in prostate cancer cells [13–17]. Panduratin A, a chalcone isolated from *Kaempferia pandurata*, induces apoptosis and G_2_/M cell cycle arrest in androgen-independent prostate cancer cells PC3 and DU145 [14]. Licochalcone A and isoliquiritigenin detected in *Glycyrrhiza glabra* inhibit proliferation and block cell cycle progression in the G_2_/M phase in PC3 and DU145 cells [15,16]. Xanthohumol identified in *Humulus lupulus* exhibited antiproliferative activities on PC3 and DU145 cells [17]. We have demonstrated that treatment of LNCaP prostate cancer cells with chalcones inhibits cell proliferation by induced cytotoxicity and apoptosis. Figure 1 presents the structures of chalcones used in this study.

**Figure 2.**
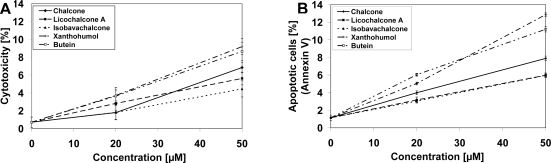
Cytotoxic and apoptotic effects of chalcones on LNCaP prostate cancer cells. The cancer cells were incubated for 48 hours with five chalcones: chalcone, licochalcone-A, isobavachalcone, xanthohumol and butein at the concentrations of 20 μM and 50 μM. The values represent mean ± SD of three independent experiments performed in quadruplicate (n = 12) for cytototoxicity, or in duplicate (n = 6) for apoptosis (p < 0.05). (A) Cytotoxic activity of chalcones in LNCaP cells. The percentage of cell death was measured by MTT cytotoxicity assay. (B) Chalcone-induced apoptosis in LNCaP cells. Detection of apoptotic cell death by annexin V-FITC staining using flow cytometry.

The LNCaP cells were incubated for 48 hours with five chalcones: chalcone, licochalcone-A, isobavachalcone, xanthohumol and butein. The chalcones inhibited growth and induced apoptosis in prostate cancer cells in a dose dependent manner. The cytotoxic effect of chalcones at the concentrations of 20–50 μM on LNCaP cells were 1.80 ± 0.82%–9.19 ± 0.87% cell death (Figure 2A). The annexin V assay revealed apoptotic cells exposed to chalcones which is demonstrated in Figure 2B (statistical analysis in Table 1). These compounds induced 3.03 ± 0.19%–12.87 ± 0.20% apoptotic cells. Our study showed that all tested chalcones exhibited low cytotoxic and apoptotic activity against LNCaP cells.

### 2.2. Cytotoxic and Apoptotic Effects of TRAIL in Prostate Cancer Cells

**Figure 3.**
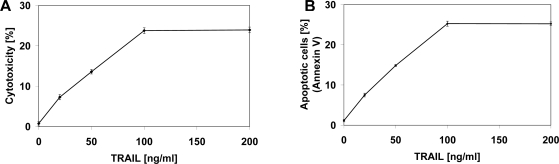
Cytotoxic and apoptotic effect of TRAIL on LNCaP prostate cancer cells. The cancer cells were incubated for 48 hours with TRAIL at the concentrations of 20–200 ng/mL. The values represent mean ± SD of three independent experiments performed in quadruplicate (n = 12) for cytototoxicity, or in duplicate (n = 6) for apoptosis (p < 0.05). (A) Cytotoxic activity of TRAIL in LNCaP cells. The percentage of cell death was measured by MTT cytotoxicity assay. (B) TRAIL-induced apoptosis in LNCaP cells. Detection of apoptotic cell death by annexin V-FITC staining using flow cytometry.

TRAIL is a naturally occurring anticancer agent expressed in immune cells that preferentially induces apoptosis in cancer cells [18,19]. Apoptosis is a highly regulated mechanism by which cells undergo programmed cell death. Resistance to apoptosis is a hallmark of cancer, with both loss of proapoptotic signals (decreased expression of death receptors TRAIL-R1 and TRAIL-R2) and the gain of the antiapoptotic mechanisms (increased expression of antiapoptotic proteins) contributing to tumorigenesis [19,20]. TRAIL induces programmed death in various cancer cells *in vitro* and *in vivo* [19,28]. We and others have shown that LNCaP prostate cancer cells are resistant to TRAIL-mediated cytotoxicity [23–25]. TRAIL induced cytotoxic and apoptotic effects in LNCaP cells in a dose-dependent manner. The cytotoxicity of TRAIL at the concentrations of 20–200 ng/mL after 48 hours’ incubation were 7.27 ± 0.64%–23.91 ± 0.70% cell death (Figure 3A). Figure 3B presents TRAIL induced apoptosis in LNCaP cancer cells determined by annexin V staining followed by flow cytometry. The 48 hours’ exposure to TRAIL increased the percentage of apoptotic cells in a dose-dependent manner to 7.50 ± 0.47%–25.23 ± 0.46%.

### 2.3. Cytotoxic and Apoptotic Effects of TRAIL in Combination with Chalcones in Prostate Cancer Cells

One of the most challenging tasks concerning cancer is to induce apoptosis in malignant cells, researchers are increasingly focusing on natural products to modulate apoptotic signaling pathways [12,27,29,30]. The role of dietary flavonoids in prevention of prostate cancer has been confirmed in previous epidemiological studies, however the mechanism of chemoprevention by these polyphenols largely remains unknown [11]. Chalcones constitute an important group of flavonoids which besides antitumor activity possess immunomodulatory properties. There is accumulating evidence that cancer prevention by flavonoids affect the anticancer immunity [27,29]. Ferguson and Philpott reported about dietary bioactive food components interacting with the immune response and having potential to reduce the risk of cancer [31]. Jakobisiak *et al.* confirmed the role of TRAIL in the immune mechanisms of protection against cancer [32]. Immunomodulation through natural substances may be considered as an alternative for prevention of malignant disease [29,31].

**Figure 4.**
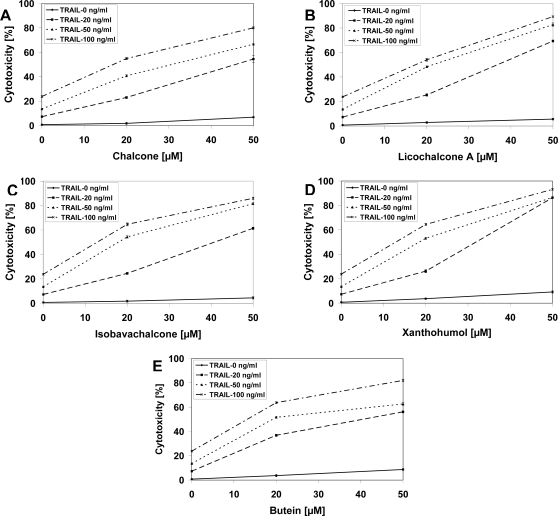
Cytotoxic activity of TRAIL in combination with chalcones in LNCaP prostate cancer cells. The cancer cells were incubated for 48 hours with TRAIL at the concentrations of 20–100 ng/mL and chalcones: (A) chalcone, (B) licochalcone-A, (C) isobavachalcone, (D) xanthohumol and (E) butein at the at the concentrations of 20 μM and 50 μM. The percentage of cell death was measured by MTT cytotoxicity assay. The values represent mean ± SD of three independent experiments performed in quadruplicate (n = 12) (p < 0.05).

As shown in Figures 2 and 3, chalcones or TRAIL alone induced little apoptotic and cytotoxic effect on LNCaP cells. We then tested chalcones in combination with TRAIL on prostate cancer cells. We investigated the cytotoxic effect on LNCaP cells of five chalcones: chalcone, licochalcone-A, isobavachalcone, xanthohumol and butein, at concentrations of 20 and 50 μM in combination with TRAIL at concentrations of 20–100 ng/mL. The cytotoxicity measured by a MTT assay is shown in Figure 4. Chalcones in combination with TRAIL increased the percentage of cell death (22.98 ± 0.84%–80.06 ± 0.87% for chalcone, 25.36 ± 0.98%–89.32 ± 0.47% for licochalcone-A, 24.47 ± 0.84%–85.90 ± 0.91% for isobavachalcone, 26.22 ± 1.22%–93.20 ± 0.60% for xanthohumol and 36.82 ± 0.87%–81.97 ± 0.84% for butein) compared to cytotoxicity of TRAIL and chalcones alone.

TRAIL and tested chalcones induced their cytotoxic effect in cancer cells by the apoptotic pathway, as the necrotic cell death percentage of LNCaP cells examined by Apoptest-FITC and lactate dehydrogenase assay was near 0%.

**Figure 5.**
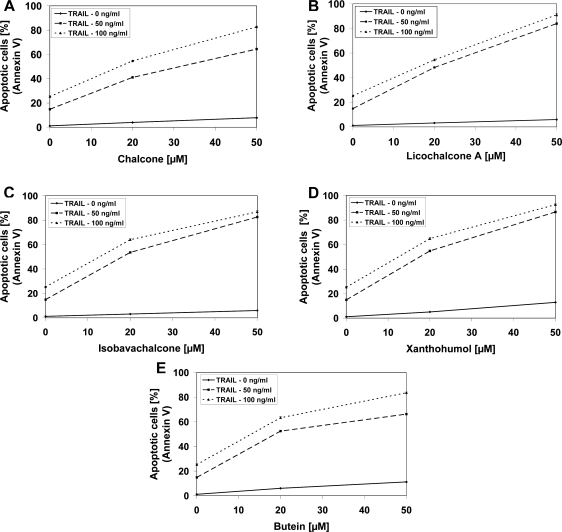
TRAIL induced apoptosis in combination with chalcones in LNCaP prostate cancer cells. The cancer cells were incubated for 48 hours with TRAIL at the concentrations of 50 ng/ml and 100 ng/mL and chalcones: (A) chalcone, (B) licochalcone-A, (C) isobavachalcone, (D) xanthohumol and (E) butein at the concentrations of 20 μM and 50 μM. Detection of apoptotic cell death by annexin V-FITC staining using flow cytometry. The values represent mean ± SD of three independent experiments performed in duplicate (n = 6) (p < 0.05).

We found out that chalcones strongly cooperated with TRAIL to induce apoptosis in LNCaP cells. The percentage of the apoptotic cells after 48 hours’ exposure to 100 ng/mL TRAIL and 50 μM chalcones were elevated at 82.71 ± 0.56% for chalcone, at 91.12 ± 1.04% for licochalcone-A, at 86.83 ± 0.85% for isobavachalcone, at 92.53 ± 0.62% for xanthohumol and at 83.53 ± 0.64% for butein (Figure 5). Statistical analysis is demonstrated in Table 2.

Chalcones enhanced the apoptosis-inducing potential of TRAIL and sensitized TRAIL-resistant LNCaP prostate cancer cells. Two similar studies with chalcones showed that isoliquiritigenin and butein synergistically induced apoptosis with TRAIL in malignant tumor cells [33,34]. Yoshida *et al.* indicated that isoliquiritigenin overcomes TRAIL-resistance in HT29 human colon cancer cells through upregulation of death receptor TRAIL-R2 (DR5) [33]. Kim explained the molecular mechanism by which butein augments TRAIL-mediated apoptosis in U937 human leukemia cells and confirmed the ability of butein to increase expression of death receptor TRAIL-R2 (DR5) and the caspase-3 activation [34]. Except butein there is no evidence of TRAIL cotreatment with other tested chalcones. The cytotoxic and apoptotic effects of chalcone, licochalcone-A, isobavachalcone and xanthohumol in combination with TRAIL on cancer cells were examined for the first time in our study. Therefore, further investigations will be required to explain the cellular signaling pathways by which chalcones sensitize cancer cells to TRAIL induced death.

Our results demonstrated that chalcones markedly augmented TRAIL mediated apoptosis in prostate cancer cells. Chalcones restored TRAIL sensitivity in TRAIL-resistant LNCaP cells. In our study all five tested chalcones: chalcone, licochalcone-A, isobavachalcone, xanthohumol and butein exhibited strong cytotoxic and apoptotic effects in combination with TRAIL against prostate cancer cells.

The different activity of chalcones in this study depends on the nature and position of substituents in the chalcone structure. The studied compounds with substituents in combination with TRAIL increased cytotoxic effect and apoptosis in comparison to chalcone. The presence of the methoxy group in xanthohumol at the 6′ position increases its activity compared to isobavachalcone. Xanthohumol alone and in combination with TRAIL was the most active compound in apoptosis induction of cancer cells (Tables 1 and 2). The isomerization of chalcones can convert their structures to flavanones. Our unpublished observations confirmed that isoxanthohumol, as an isomerization product of xanthohumol, showed with or without TRAIL lower cytotoxic and apoptotic effect against LNCaP cells than chalcone (without substituents). It may indicate the important role of the basic structure of chalcone in cytotoxic and apoptotic reactions.

Many plant and animal extract rich in phenols and polyphenols shown various biological activities including antioxidant, anticancer and immunomodulatory properties [35–38]. TRAIL is a key effector molecule expressed on immune cells responsible for surveillance against tumor development [32]. Our study showed the impact of chalcones on the anticancer immune defense through the interaction and modulation of the TRAIL-mediated apoptotic pathway in prostate cancer cells. The findings suggest that chalcones may exert a chemopreventive effect in cooperation with endogenous TRAIL *in vivo*. The TRAIL potential enhancement by chalcones suggests that these compounds can be used in prostate cancer chemoprevention.

## 3. Experimental Section

### 3.1. Chemicals

#### 3.1.1. Chalcones

The five chalcones: chalcone (*trans*-benzylideneacetophenone) (**1**), licochalcone-A (E-3-[5-(1,1-diethyl-2-propenyl)-4-hydroxy-2-methoxyphenyl]-1-(4-hydroxypenyl)-2-propen-1-one) (**2**), isobava--chalcone (2′,4′,4-trihydroxy-3′-[3′-methylbut-3′-ethyl]chalcone) (**3**), xanthohumol (2′,4,4′-trihydroxy-3′-prenyl-6′-methoxychalcone) (**4**) and butein (2′,3,4,4′-tetrahydroxychalcone) (**5**) were purchased from Alexis Biochemicals (Lausanne, Switzerland).

#### 3.1.2. TRAIL

Recombinat human TRAIL was purchased from PeproTech Inc. (Rocky Hill, NJ, USA).

### 3.2. Cell Culture

The experiments were performed on human hormone-sensitivity prostate cancer LNCaP cells (DSMZ - German Collection of Microorganisms and Cell Cultures, Braunschweig, Germany). The cancer cells were grown in monolayer cultures in RPMI 1640 medium with 10% fetal bovine serum, 4 mM l-glutamine, 100 U/mL penicillin, and 100 μg/mL streptomycin and incubated at 37 °C in atmosphere containing 5% CO_2_ [24,25]. Reagents for cells culture were purchased from PAA The Cell Culture Company (Pasching, Austria).

### 3.3. Cytotoxicity Assay

The cytotoxicity was measured by a 3-[4,5-dimethylthiazol-2-yl]-2,5 diphenyltetrazolium (MTT) assay as described [24,25,28]. The LNCaP cells (2 × 10^5^/mL) were seeded 48 hours before the experiments in a 96-well plate. Various combinations of chalcones (20 μM and 50 μM) with or without TRAIL (20–200 ng/mL) were added to the cells, and 48 hours later the medium was removed and 20 μL MTT solutions (5 mg/mL) (Sigma Chemical Company, St Louis, MO, USA) were added to each well for 4 h. The resulting crystals were dissolved in DMSO. Controls included native cells and medium alone. The spectrophotometric absorbance of each well was measured using a microplate reader (ELx 800, Bio-Tek Instruments Inc., Winooski, VT, USA) at 550 nm. The percent cytotoxicity was calculated by the formula: percent cytotoxicity (cell death) = (1 − [absorbance of experimental wells/absorbance of control wells]) × 100%.

### 3.4. Lactate Dehydrogenase Release Assay

Lactate dehydrogenase (LDH) is a stable cytosolic enzyme that is released upon membrane damage in necrotic cells. LDH activity was measured using a commercial cytotoxicity assay kit (Roche Diagnostics GmbH, Mannheim, Germany), in which LDH released in culture supernatants is measured with a coupled enzymatic assay, resulting in conversion of a tetrazolium salt into red formazan product. The LNCaP cells were treated with various concentrations of chalcones (20 μM and 50 μM) alone and in combination with TRAIL (20–200 ng/mL) for the indicated period of time. The sample solution (supernatant) was removed and LDH released from cells was measured in culture medium. The maximal release was obtained after treating control cells with 1% Triton X-100 (Sigma Chemical Company, St. Louis, MO) for 10 minutes at a room temperature [24,25,28]. The necrotic percentage was expressed using the formula, (sample value/maximal release) × 100%.

### 3.5. Determination of Apoptotic Cell Death by Annexin V-FITC Staining

Apoptosis was measured using flow cytometry to quantify the levels of decentable phosphatidylserine (PS) on the outer membrane of apoptotic cells. Externalized PS on the outer surface of the cytoplasmic membrane becomes labeled by fluorescein labeled annexin V, which has a high affinity for PS-containing phospholipids bilayers. Prostate cancer cell line LNCaP (2 × 10^5^/mL) cells were seeded in 24-well plates for 48 hours and then exposed to chalcones (20 μM and 50 μM) and/or TRAIL (20–200 ng/mL) for 48 hours. After a 48 hour incubation cancer cells were washed twice with PBS and resuspended in 1 mL of binding buffer. Five hundred microliters of cell suspension was then incubated with 5 μL of annexin V-FITC and 10 μL of propidium iodide (PI) for 10 min at a room temperature in the dark. Annexin V assay was performed using the Apoptotest-FITC Kit (Dako, Glostrup, Denmark). The population of annexin V-positive cells was evaluated by flow cytometry (BD FACScan, Becton Dickinson Immnunocytometry Systems, San Jose, CA, USA) [25,35].

### 3.6. Statistical Analysis

The results are expressed as means ± S.D. obtained from three separate experiments performed in quadruplicates (n = 12) for cytototoxicity or duplicates (n = 6) for apoptosis. The experimental means were compared to the means of untreated prostate cancer cells harvested in a parallel manner and the data was polled for replicate experiments. Statistical significance was evaluated using one- and multiple-way ANOVA or Kruskal-Wallis test followed by the post hoc test. p-values < 0.05 were considered significant.

## 4. Conclusions

In this work we investigated the apoptotic and/or cytotoxic effect of five chalcones (chalcone, lipochalcone-A, isobavachalcone, xanthohumol and butein) on prostate cancer cells in combination with TRAIL. Our findings indicated that all tested chalcones: chalcone, lipochalcone-A, isobavachalcone, xanthohumol and butein markedly augment TRAIL mediated apoptosis in LNCaP cells. We showed for the first time that chalcones sensitize prostate cancer cells to TRAIL induced apoptosis. The obtained results suggest that chalcones help anticancer immune defense in which endogenous TRAIL takes part. The TRAIL-mediated cytotoxic and apoptotic pathways may be a target of the chemopreventive agents in prostate cancer cells and the overcoming TRAIL-resistance by chalcones may be one of the mechanisms responsible for their cancer preventive effects.

## Figures and Tables

**Table 1.** Apoptosis induced by chalcones - p values.

**Post-hoc test**											
**p=**	**(1)**	**(2)**	**(3)**	**(4)**	**(5)**	**(6)**	**(7)**	**(8)**	**(9)**	**(10)**	**(11)**
**Control: LNCaP cells (1)**		2E-05	1E-05	8E-06	1E-05	2E-05	3E-05	3E-05	2E-05	1E-05	2E-05
**Chalcone 20 [μM] (2)**	2E-05		2E-05	2E-05	8E-06	8E-06	2E-05	9E-06	3E-05	2E-05	3E-05
**Chalcone 50 [μM] (3)**	1E-05	2E-05		3E-05	2E-05	1E-05	8E-06	2E-05	2E-05	9E-06	9E-06
**Licochalcone-A 20 [μM] (4)**	8E-06	2E-05	3E-05		2E-05	0.1632	2E-05	8E-06	1E-05	3E-05	1E-05
**Licochalcone-A 50 [μM] (5)**	1E-05	8E-06	2E-05	2E-05		3E-05	0.8617	2E-05	2E-05	0.5419	8E-06
**Isobavachalcone 20 [μM] (6)**	2E-05	8E-06	1E-05	0.1632	3E-05		2E-05	2E-05	1E-05	3E-05	1E-05
**Isobavachalcone 50 [μM] (7)**	3E-05	2E-05	8E-06	2E-05	0.8617	2E-05		9E-06	2E-05	0.7127	2E-05
**Xanthohumol 20 [μM] (8)**	3E-05	9E-06	2E-05	8E-06	2E-05	2E-05	9E-06		3E-05	8E-06	2E-05
**Xanthohumol 50 [μM] (9)**	2E-05	3E-05	2E-05	1E-05	2E-05	1E-05	2E-05	3E-05		8E-06	9E-06
**Butein 20 [μM] (10)**	1E-05	2E-05	9E-06	3E-05	0.5419	3E-05	0.7127	8E-06	8E-06		2E-05
**Butein 50 [μM] (11)**	2E-05	3E-05	9E-06	1E-05	8E-06	1E-05	2E-05	2E-05	9E-06	2E-05	

**Table 2.** Apoptosis induced by chalcones at concentrations 20 μM and 50 μM with TRAIL at concentrations 50 ng/mL and 100 ng/mL–p values.

**Post - hoc test.**	**TRAIL - 50 ng/ml**									
	**Chalcones 20 μM**					**Chalcones 50 μM**				
**p=**	**(1)**	**(2)**	**(3)**	**(4)**	**(5)**	**(1)**	**(2)**	**(3)**	**(4)**	**(5)**
**Chalcone (1)**		0.000112	0.000161	0.000129	0.00012		0.000161	0.00012	0.000129	0.000112
**Licochalcone (2)**	0.000112		0.00012	0.000161	0.000112	0.000161		0.000112	0.000112	0.00012
**Isobavachalcone (3)**	0.000161	0.00012		0.000126	0.000257	0.00012	0.000112		0.00012	0.000112
**Xanthohumol (4)**	0.000129	0.000161	0.000126		0.00012	0.000129	0.000112	0.00012		0.000161
**Butein (5)**	0.00012	0.000112	0.000257	0.00012		0.000112	0.00012	0.000112	0.000161	
	**TRAIL - 100 ng/ml**									
	**Chalcones 20 μM**					**Chalcones 50 μM**				
**p=**	**(1)**	**(2)**	**(3)**	**(4)**	**(5)**	**(1)**	**(2)**	**(3)**	**(4)**	**(5)**
**Chalcone (1)**		0.402463	0.00012	0.000161	0.000112		0.000161	0.00012	0.000129	0.010564
**Licochalcone (2)**	0.402463		0.000161	0.000129	0.00012	0.000161		0.000112	0.000138	0.00012
**Isobavachalcone (3)**	0.00012	0.000161		0.00315	0.019365	0.00012	0.000112		0.00012	0.000112
**Xanthohumol (4)**	0.000161	0.000129	0.00315		0.000122	0.000129	0.000138	0.00012		0.000161
**Butein (5)**	0.000112	0.00012	0.019365	0.000122		0.010564	0.00012	0.000112	0.000161	
